# Efficacy and Safety of Dual Antiplatelet Therapy with the Routine Use of Prasugrel for Flow Diversion of Cerebral Unruptured Aneurysms

**DOI:** 10.1007/s00062-023-01355-2

**Published:** 2023-10-17

**Authors:** Kenichiro Suyama, Ichiro Nakahara, Shoji Matsumoto, Jun Morioka, Jun Tanabe, Akiko Hasebe, Sadayoshi Watanabe

**Affiliations:** Department of Comprehensive Strokology, Fujita Health University School of Medicine, 1–98 Dengakugakubo, Kutsukake-cho, Toyoake, Aichi Japan

**Keywords:** Intracranial Aneurysm, Antiplatelet Drug, Prasugrel Hydrochloride, Endovascular Procedures

## Abstract

**Purpose:**

Prasugrel is not approved for patients treated with flow diverters, which have a high metal coverage ratio. However, robust antiplatelet therapy with prasugrel may prevent thromboembolic complications. We administered prasugrel and aspirin to all patients treated with flow diverters and reported the safety of the antiplatelet therapy regimen.

**Methods:**

This retrospective, single-center study evaluated the angiographic and clinical data of consecutive patients treated with flow diverters for cerebral unruptured aneurysms between June 2020 and May 2022. All patients received dual antiplatelet therapy, including prasugrel and aspirin. The administration of prasugrel ended 3 or 6 months after the procedure, whereas aspirin use continued for at least 12 months. Periprocedural complications (< 30 days post-procedure) and delayed complications (> 30 days post-procedure) were recorded.

**Results:**

During the study period, 120 unruptured aneurysms were treated with flow diverters in 110 patients. All patients, except one, survived longer than 12 months after the procedure. The rate of thromboembolic complications was 6.4%, and more than half of the patients had transient symptoms; one (0.9%) had a major ischemic stroke. One patient (0.9%) each had an asymptomatic, small subarachnoid hemorrhage and significant hemorrhagic complications with melena. The rate of permanent neurological deficits was 1.8%, and the mortality rate was 0.9%.

**Conclusions:**

Dual antiplatelet therapy comprising routine use of prasugrel and aspirin for flow diverter-implanted patients possibly contributed to a low rate of thromboembolic complications and low risk of hemorrhagic complications.

## Introduction

Flow diverters (FD) have a high metal coverage ratio, and dual antiplatelet therapy comprising aspirin (ASA) and clopidogrel (CPG) during the perioperative period is standard for preventing ischemic complications. However, some individuals have genetic variations that cause a failed response to CPG [1–4]. Patients resistant to CPG are at higher risk for ischemic complications [5–7]. The frequency of resistance to CPG is higher among Asians (18–23% and up to 70%) than among Caucasians (3%) [8]. Prasugrel (PSG) is a new-generation P2Y12 receptor antagonist, has less varied interindividual effects than CPG, and is widely used for coronary interventions [9–11]. However, the use of PSG is limited in cerebrovascular diseases because of the high risk of bleeding events and the lack of clinical data [10, 12–14]. If a safe dosage is identified, PSG can be administered to all patients, irrespective of their genetic variations. We previously reported the stratified use of PSG at the time of endovascular treatment [15]. In this study, all patients treated with FD received dual antiplatelet therapy comprising PSG and ASA. Herein, we report the safety of our antiplatelet therapy regimen.

## Methods

### Patient Selection

This single-center, retrospective study evaluated the angiographic and clinical data of consecutive patients treated with FD for unruptured cerebral aneurysms from June 2020 to May 2022. The FDs used during this study were Pipeline Shield (Medtronic, Irvine, CA, USA) and FRED (MicroVention-Terumo, Tustin, CA, USA). The treatment indications for FD for cerebral aneurysms were as follows: maximum dome diameter larger than 5 mm and wide-neck (neck width of 4 mm or dome-neck ratio < 2), saccular, or fusiform intracranial unruptured aneurysms. Patients with ruptured aneurysms and patients with aneurysms at extracranial internal carotid artery or extracranial vertebral artery were excluded. Data were obtained from the medical charts and retrospectively reviewed. Baseline characteristics, including demographics, medical history, and aneurysm characteristics, were recorded. Treatment characteristics, including the procedure time, adjunctive coil, and type and number of FD, were collected. All intraprocedural, periprocedural, and delayed complications were reported.

This study was approved by the Institutional Ethics Committee (approval number: HM 22-125). The need for written informed consent was waived because the option to opt out of the study was posted on the institutional website and as the study had a retrospective design and involved the analysis of routine follow-up data.

### Perioperative Antiplatelet Management

Before FD placement, all patients received dual antiplatelet therapy with CPG (75 mg) and ASA (100 mg) for 12–14 days. The P2Y12 reaction unit (PRU) and aspirin reaction unit (ARU) were monitored using the VerifyNow assay (Accumetrics, San Diego, CA, USA) 2 days before the procedure, and all patients were switched from CPG to PSG. Patients with PRU > 210 received a 20 mg loading dose of PSG, followed by a 3.75 mg/day, while those with PRU of 60–210 mg received 3.75 mg/day of PSG. Patients with PRU < 60 were administered 1.875 mg/day of PSG. The PRU was re-examined at 4 days and 3 months after the procedure to check the effectiveness of PSG.

Angiographic follow-up was performed at 3, 6, and 12 months after the procedure. PSG was discontinued for 6 months after the procedure, and ASA was continued for at least 12 months. In older patients or those with low body weight, if the follow-up angiogram at 3 months post-procedure showed complete occlusion, PSG was discontinued for 3 months.

As PSG use to treat cerebral aneurysms was off-label, the institutional off-label use committee approved this use. In addition, we described the use of off-label antiplatelet agents in the individual consent form for endovascular treatment.

### Endovascular Treatment

All procedures were performed under general anesthesia with systemic heparinization to maintain an activated clotting time of more than 250 s. A transfemoral approach was used for all patients. Aneurysms larger than 15 mm without intra-aneurysmal thrombosis were treated with additional coils. Coils were also used for aneurysms with bleb or high dome/neck ration, even those with a diameter smaller than 15 mm. After FD deployment, high-resolution cone-beam computed tomography was performed to evaluate the wall apposition of the FD, and post-dilatation with a balloon catheter was performed for incomplete apposition.

### Complications

The primary outcome was the rate of complications. Periprocedural complications were defined as those occurring within 30 days after the procedure. The following periprocedural complications were evaluated: symptomatic thromboembolic complications (TECs), intracranial hemorrhage, symptomatic hemorrhagic complications, and all symptomatic complications. Symptomatic TECs were defined as a diffusion-weighted imaging (DWI)-positive image with neurological findings that developed within 30 days after the procedure [15]. Intracranial hemorrhages included asymptomatic hemorrhages. Symptomatic hemorrhagic complications were defined according to the International Society for Thrombosis and Hemostasis (ISTH) major bleeding criteria [16].

Delayed complications were the symptomatic complications that occurred more than 30 days after the procedure. The neurological findings were evaluated by two neurosurgeons at follow-up angiogram admission, and all evaluators had more than 10 years of experience. PSG was administered for 3 or 6 months after the procedure, and patients received single-antiplatelet therapy comprising ASA for at least 12 months.

### Magnetic Resonance Imaging

Magnetic resonance imaging, including DWI, T2-weighted imaging, fluid-attenuated inversion recovery, and magnetic resonance angiography without contrast media, was performed for all patients within 48 h after the procedure. We previously proposed the following DWI grading scale [17]: grade A, no high-intensity area (HIA); grade B, small HIAs (≤ 5 spots and each ≤ 10 mm); grade C, some small HIAs (> 5 spots and each ≤ 10 mm); and grade D, large HIAs (≥ 1 spot > 10 mm). The patients were classified into four groups based on positive DWI findings.

### Statistical Analysis

Data are presented as the mean ± standard deviation or median and interquartile range for continuous variables and as frequencies for categorical variables. The statistical analysis was performed using the Student *t*-, Mann-Whitney *U*, or Fisher exact tests. Statistical significance was set at *P* < 0.05. Statistical analyses were performed using EZR software.

## Results

During the study period, 120 unruptured aneurysms in 110 patients were treated with FD at our institute. Patient characteristics and aneurysms are presented in Table 1. The median maximum dome diameter was 7.1 mm, and the median neck size was 4.9 mm. While 23% (*n* = 28) of the aneurysms were large (≤ 10–25 mm), 4.2% (*n* = 5) were giant (≤ 25 mm). Sixteen (13.3%) of the 120 aneurysms were recurrent aneurysms after previous treatment. Fourteen recurred after endovascular treatment (coil embolization, 10; stent-assisted coil embolization, 2; flow diverter, 2) and two after clipping. The results of the procedure are listed in Table 2.

**Table 1:** Baseline characteristics of the cohort

Characteristics	Value
No. of patients	110
No. of aneurysms	120
Age, years	56.0 ± 13.8
Sex (Female)	84 (76.4%)
Aneurysm size, mm	7.1 (5.7–10.3)
Neck, mm	4.9 (3.8–7.3)
**Location**	
ICA cavernous	14 (11.7%)
ICA paraclinoid	65 (54.2%)
ICA supraclinoid	11 (9.2%)
MCA	4 (3.3%)
ACA	2 (1.7%)
VA	21 (17.5%)
BA	3 (2.5%)
*Retreatment after previous treatment*	16 (13.3%)

*ICA* internal carotid artery, *MCA* middle cerebral artery, *ACA* anterior cerebral artery, *VA* vertebral artery, *BA* basilar artery

**Table 2:** Description of the procedures performed in the cohort

Characteristics	Value
Procedure time, min (*n* = 110)	66.5 (52.3–93.8)
Additional coil (*n* = 120)	16 (13.3%)
Posdilatation with balloon (*n* = 110)	64 (58.2%)
*Type of FD (n* *=* *110)*	
PED	44 (40.0%)
FRED	66 (60.0%)
*No. of FD (n* *=* *110)*	
1	99 (90.0%)
≥ 2	11 (10.0%)

*FD* flow diverter, *PED* Pipeline, *FRED* Flow Re-direction Endoluminal Device

**Fig. 1:**
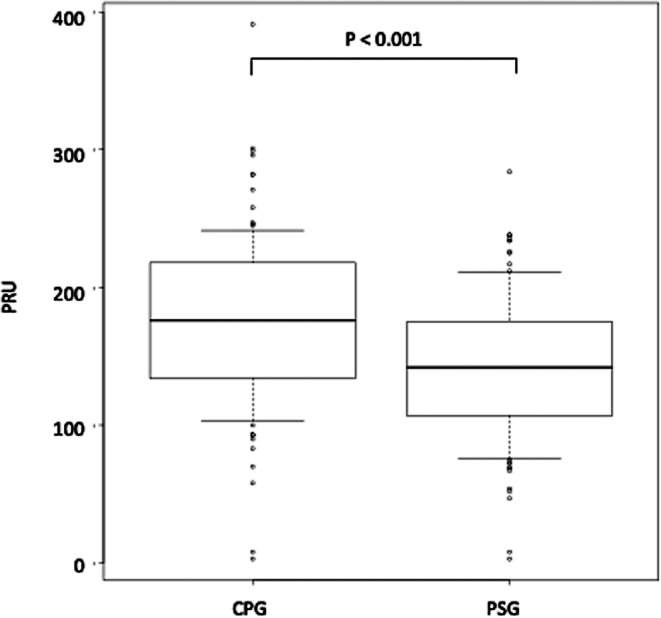
PRU comparison. PRU measured 2 days before the procedure with CPG was compared with that measured 4 days after the procedure with PSG (176.8 ± 62.0 vs. 142.5 ± 51.4, *P* < 0.001). *PRU* P2Y12 reaction unit, *CPG* clopidogrel, *PSG* prasugrel

**Fig. 2:**
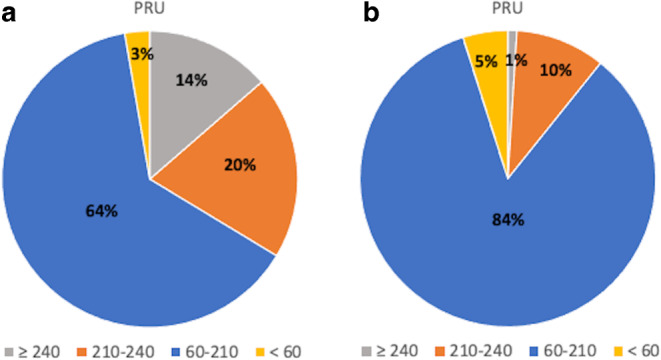
Distribution of PRU. The PRU distribution **a** 2 days before the procedure with CPG and **b** 4 days after the procedure with PSG. *PRU* P2Y12 reaction unit, *CPG* clopidogrel, *PSG* prasugrel

The PRU with the administration of PSG at 4 days after the procedure was significantly decreased compared to that with the administration of CPG at 2 days before the procedure (142.5 ± 51.4 vs. 176.8 ± 62.0; *P* < 0.001) (Fig. 1). The PRU at 3 months after the procedure did not significantly differ from that at 4 days after the procedure (121.0 ± 55.7 vs. 142.5 ± 51.4; *P* = 0.13). The distribution of the PRU at 2 days before the procedure with CPG and at 4 days after the procedure with PSG is presented in Fig. 2. Complications are presented in Table 3. Catheter-induced dissection was recorded in two patients; both were asymptomatic and did not need additional treatment. Symptomatic TECs were observed in seven patients (6.4%). While 4/7 patients had transient symptoms, which improved, 2/7 had a minor stroke, and another patient had a major stroke with repeated in-stent thrombosis resulting in death after the procedure. An intracranial hemorrhage occurred in only one patient with a small asymptomatic subarachnoid hemorrhage detected by postprocedural computed tomography. Symptomatic hemorrhagic complications meeting the ISTH criteria for major bleeding occurred in one patient with Osler disease. This patient had anemia of unknown cause that was improved by a blood transfusion of red blood cells, and the patient left the hospital 4 days after the procedure with no neurological deficits. Five patients had other mild complications, including inguinal subcutaneous hematoma in three patients, transient diplopia in one patient, and contrast media allergy in one patient. The rate of all symptomatic complications during the periprocedural period was 12.7%, while that of permanent neurological deficits was 1.8% (*n* = 2) for those with a minor ischemic stroke. The mortality rate was 0.9% (*n* = 1).

**Table 3:** Complications reported in the cohort

Complications	Value (*n* = 110)
**Periprocedural complications**	
Symptomatic thromboembolic complications	7 (6.4%)
Intracranial hemorrhage	1 (0.9%)
Symptomatic hemorrhagic complications	1 (0.9%)
All symptomatic complications	14 (12.7%)
**Delayed complications**	1 (0.9%)

**Fig. 3:**
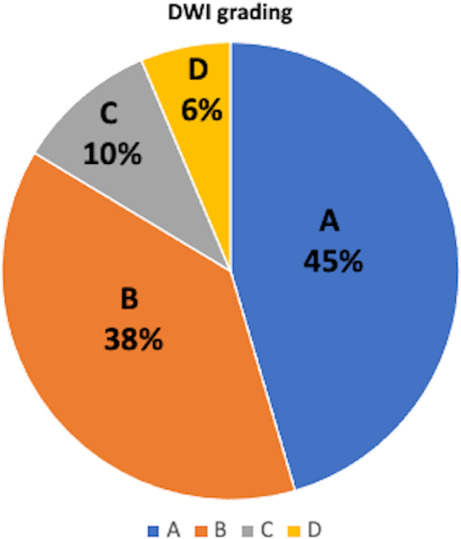
Distribution of DWI grading. Grade A, no high-intensity areas (HIAs); Grade B, small HIAs (≤ 5 spots and each ≤ 10 mm); Grade C, some small HIAs (> 5 spots and each ≤ 10 mm); and Grade D, large HIAs (at least one spot > 10 mm). *DWI* diffusion-weighted imaging, *HIA* high-intensity area

DWI was performed for all patients. The DWI grading frequency is shown in Fig. 3. The most common grade was grade A (45%), followed by grades B (38%), C (10%), and D (6%).

The mean follow-up period was 25.5 ± 6.3 months, and all patients except one survived more than 12 months after the procedure. One patient died within 12 months of follow-up. Delayed hemorrhagic complications occurred more than 30 days after the procedure in one patient, who developed melena 2 months after the procedure and was treated using an endoscope. No delayed ischemic complications were observed during the follow-up period.

A comparison of the clinical features of patients with and without TECs is shown in Table 4. The procedure time was significantly longer for patients with TECs than those without TEC (98 vs. 65 min; *P* < 0.01). Furthermore, more patients with TECs required treatment with additional coiling than those without TEC (57.1% vs. 11.7%; *P* < 0.01). The type of FD, use of multiple FDs, and PRU and ARU values were not significantly different between groups. TECs were associated with lengthy and complex procedures. A multivariate analysis was not performed, given the small number of patients with TECs.

**Table 4:** Intergroup comparison of participant characteristics in the TEC and no-TEC groups

Characteristics	TEC (*n* = 7)	No TEC (*n* = 103)	*P*-value
Age	59.0 ± 11.4	55.8 ± 14.0	0.56
Posterior circulation	1 (14.3)	23 (22.3)	1
Aneurysm size, mm	6.7 (5.6–13.8)	7.4 (6.1–11.1)	0.94
Procedure time, min	98 (87–194.0)	65 (52–88.5)	0.003
Type of FDs (FRED)	4 (57.1)	62 (60.2)	1
Multiple FDs	1 (14.3)	11 (10.7)	0.57
Adjunctive coil embolization	4 (57.1)	12 (11.7)	0.008
PRU 2 days before the procedure	155.3 ± 57.4	178.2 ± 62.3	0.35
PRU 4 days after the procedure	176.3 ± 23.0	141.1 ± 51.8	0.18
ARU	480.4 ± 74.8	465.6 ± 80.5	0.64

*TEC* thromboembolic complication, *FD* flow diverter, *PRU* P2Y12 reaction unit, *ARU* aspirin reaction unit

## Discussion

In neurointervention, PSG is effective for patients who are resistant to CPG [12–15]. This study is novel because PSG was administered to all patients, not just those with inadequate response to CPG. Another important characteristic of this study is that the modality of endovascular treatment for cerebral aneurysms was limited to FD. During previous studies on the efficacy of PSG, endovascular treatment for cerebral aneurysms included various modalities, such as coil embolization, stent-assisted coil embolization, and FD [12–15, 18–21]. The FD has a high metal coverage ratio, and conventional antiplatelet therapy may be insufficient. Therefore, robust antiplatelet therapy with PSG may effectively prevent ischemic complications.

The use of PSG has been limited in neurointervention because of the high risk of hemorrhagic complications [10, 12]. Globally, the loading dose of PSG is 60 mg, and the daily maintenance dose is 10 mg. However, previous studies [10, 12] have reported a high risk of hemorrhagic complications with PSG at this dose. Some studies recently reported decreased TECs with no increase in hemorrhagic complications with low-dose PSG [15, 18–21]. PSG was administered at a loading dose of 20 mg and a maintenance dose of 5 mg/day in those studies. In this study, we administered a loading dose of 20 mg and a maintenance dose of 3.75 mg/day. These doses were similar to those administered in a coronary intervention study on elderly Japanese patients with low body weight [22]. Moreover, the 3.75 mg/day dose of PSG has been reported to prevent ischemic stroke recurrence and has been approved by the Japanese government based on the clinical trial results [23]. In this study, only one patient (0.9%) had an intracranial hemorrhage, and another patient (0.9%) had major symptomatic hemorrhagic complications, both of which were not life-threatening. Delayed hemorrhagic complications were also observed in only one patient (0.9%) with melena. Thus, the rate of hemorrhagic complications was very low.

We previously reported the stratified use of PSG when performing an endovascular treatment, including coil embolization, stent-assisted coil embolization, and FD, and the rate of TECs was 6.6% [15]. The rate of TECs in this study (6.4%) is considered equivalent to that associated with the stratified use of PSG. While in our previous study, the most common DWI grade was grade B (37%), followed by grades C (33%), A (20%), and D (9%) [15], in this study, the most frequent DWI grade was grade A (45%), followed by grades B (38%), C (10%), and D (6%), in this study. Nevertheless, the treatment modality was limited to FD with a high metal coverage ratio and a tendency for ischemic complications; the frequency of grade A without DWI observations of HIAs was significantly higher than that in our previous study [15]. However, the two studies had different characteristics, and the PSG dose might be effective for reducing TECs without increasing the risk of hemorrhagic complications.

In this study, major ischemic stroke and intracranial hemorrhage were recorded in only one patient (0.9%) each. One of the reasons for the low major complication rate may be the size of the treated aneurysms as well as PSG use. The median maximum dome diameter in this study was 7.1 mm, and the rate of aneurysms with dome diameter of less than 10 mm was 72.5%. Complication rates are similar to reports of FD for small aneurysms [24, 25].

There were several reasons for administering PSG to all patients. In our previous study, PSG was administered to patients resistant to CPG, determined by measuring the PRU and using a cutoff value ≥ 240 [15]. Studies have reported different PRU cutoff values for CPG resistance [26, 27]. Tan et al. have reported a cutoff value of 208 [27]. However, we used the cutoff value ≥ 240 based on the study by Delgado et al. [26]. In this study, 20% of the patients had PRU values ranging from 208 to 240, and when the cutoff value of PRU was set to 240, these patients were not administered PSG and were likely to experience TECs. All patients were administered PSG to gain sufficient antiplatelet reactivity.

Patients with CYP2C19 phenotypes were divided into extensive, intermediate, and poor metabolizers [28]. Poor and intermediate metabolizers were at significantly high risk for stent thrombosis after percutaneous coronary intervention [29]. The administration of PSG significantly reduced the PRU in the intermediate and poor metabolizers [30] but not in the extensive metabolizers. Considering these factors, PSG may effectively prevent TECs in not only the poor metabolizers but also the intermediate metabolizers when treated with FDs, which have a high metal coverage ratio. The rate of intermediate and poor metabolizers of phenotypes in Japan is very high (63%) [28]. Therefore, if a safe dosage is identified, it is reasonable to administer PSG to all patients treated with FD. Another reason for administering PSG to all patients is the accuracy of PRU. Since a pharmacogenetic analysis to distinguish the phenotypes requires time and financing, we used the PRU to identify CPG-resistant patients instead. However, PRU correlates with phenotype and platelet inhibition, the correlation is not perfect [31], potentially overlooking CPG-resistant patients.

When all patients received dual antiplatelet therapy comprising PSG and ASA, the purpose of measuring the PRU was to detect hyper-responders to PSG. In this study, the rate of hyper-responders to a PRU < 60 was 5%. Therefore, it is important to safely administer PSG to all patients to detect hyper-responsiveness and reduce the dose of PSG.

Ticagrelor is a reversible inhibitor of P2Y12 of the thienopyridine class and its efficacy in FD treatment has been reported [32, 33]. Ticagrelor does not require hepatic metabolization for activation, patients with genetic resistance to clopidogrel due to alterations in the CYP2C19 enzyme are not equally resistant to ticagrelor [34]. The advantages of ticagrelor are its rapid action and reversibility whereas a disadvantage is the twice-daily dosing. In Japan, PSG has been approved for the prevention of ischemic stroke by the Japanese government whereas ticagrelor has not been approved for cerebrovascular disease due to insufficient evidence. In this study, we used only PSG and not ticagrelor. No study has directly compared prasugrel to ticagrelor, and further studies are necessary to find optimal antiplatelet therapy for FD.

Recently, some studies have reported the efficacy and safety of single antiplatelet therapy comprising PSG with surface-modified FDs, such as the Pipeline Shield FD, p64 MW hydrophilic polymer-coated FD, and p48 MW hydrophilic polymer-coated FD [35–40]. However, these were pilot studies with small sample sizes. The standard periprocedural antiplatelet therapy with FD is dual antiplatelet therapy. Based on the results of this study, we expect that dual antiplatelet therapy comprising PSG will become the standard treatment with FD.

In this study, we changed the treatment with CPG to PSG. We did not use PSG from the beginning to observe the change in the PRU when we switched from CPG to PSG. We also started using an antiplatelet therapy regimen by administering PSG from the beginning and plan to report those results in the future.

This study has several limitations. First, it was not a randomized, controlled study, and the complication rate was compared with another study performed at our institute that used an identical definition of complications. A prospective, randomized, controlled study could more objectively elucidate the efficacy and superiority of PSG with FDs compared to CPG. Second, most of the patients in this study were Japanese; therefore, the reported PSG dose might not apply to Caucasians.

## Conclusions

Dual antiplatelet therapy comprising the routine use of low-dose PSG for FD effectively achieves antiplatelet reactivity for the prevention of TECs, is safe, and is associated with a low risk of hemorrhagic complications. Based on our findings, we expect that dual antiplatelet therapy comprising prasugrel could potentially be a standard treatment with FD.

## Abbreviations

ARU: aspirin reaction unit
ASA: aspirin
CPG: clopidogrel
DWI: diffusion-weighted imaging
FD: Flow diverter
HIA: high-intensity area
PRU: P2Y12 reaction unit
PSG: prasugrel
TEC: thromboembolic complication
